# The Immunomodulator VacA Promotes Immune Tolerance and Persistent *Helicobacter pylori* Infection through Its Activities on T-Cells and Antigen-Presenting Cells

**DOI:** 10.3390/toxins8060187

**Published:** 2016-06-16

**Authors:** Aleksandra Djekic, Anne Müller

**Affiliations:** Institute of Molecular Cancer Research, University of Zürich, Winterthurerstr. 190, 8057 Zürich, Switzerland

**Keywords:** persistent infection, myeloid cells, professional antigen-presenting cells, T-cell priming, effector T-cells, regulatory T-cells, immune tolerance

## Abstract

VacA is a pore-forming toxin that has long been known to induce vacuolization in gastric epithelial cells and to be linked to gastric disorders caused by *H. pylori* infection. Its role as a major colonization and persistence determinant of *H. pylori* is less well-understood. The purpose of this review is to discuss the various target cell types of VacA and its mechanism of action; specifically, we focus on the evidence showing that VacA targets myeloid cells and T-cells to directly and indirectly prevent *H. pylori*-specific T-cell responses and immune control of the infection. In particular, the ability of VacA-proficient *H. pylori* to skew T-cell responses towards regulatory T-cells and the effects of Tregs on *H. pylori* chronicity are highlighted. The by-stander effects of VacA-driven immunomodulation on extragastric diseases are discussed as well.

## 1. VacA Has Immunomodulatory and -Suppressive Activity on Various Cell Types of the Mammalian Immune System that Ensure the Persistence of *Helicobacter pylori* in Its Host

The immunomodulatory and colonization-promoting activity of the vacuolating cytotoxin VacA of *H. pylori* was discovered more than a decade after its vacuolating activity on epithelial cells and remains much less well-understood. A study published in 2001 provided the first experimental evidence for a critical role of VacA in mouse stomach colonization: VacA mutants were shown to be outcompeted by the parental wild type strains in mixed infections and exhibited an ID_50_ that was more than two orders of magnitude higher than that of the corresponding wild types in single infections [1]. This finding has since been confirmed and extended in single infections with isogenic VacA null mutants in the same or related strain backgrounds; one study found VacA mutants to colonize at significantly lower levels [2], whereas, in another study, VacA mutants were recovered from <20% of infected mice (relative to >90% for wild type) and at lower densities [3]. In humans, the majority of isolates express some form of VacA, from alleles that vary substantially in sequence and expression level. A study of 43 independent isolates—the most comprehensive study to date—revealed that all were positive for the *vacA* gene, but only 28 (65%) expressed the corresponding protein and exhibited vacuolating activity on HeLa cells *in vitro* [4]. Interestingly, genetic manipulation of the s and i regions (*i.e.*, regions that are highly polymorphic in human isolates) affects the ability of *H. pylori* strains to infect mice; the s2i2 allele of VacA appears to promote murine colonization, whereas the (highly expressed and highly cytotoxic) s1i1 allele does not [3]. This finding is particularly interesting, as the s1i1 allele of VacA has been linked to gastric cancer and premalignant lesions in several studies [3,5,6]. One important conclusion from the combined epidemiological and experimental studies is thus that the cytotoxic, tissue damage-inducing properties and the immunomodulatory properties of VacA are likely genetically and functionally distinct; s2i2-expressing strains exhibit a clear phenotype upon deletion of their *vacA* allele despite it encoding a non-cytotoxic version of the protein. It must be noted, however, that VacA expression is not an absolute requirement for stomach colonization. Not only have human isolates been identified that lack VacA expression as mentioned above [4], but other related *Helicobacter* species such as *H. felis* and *H. mustelae* exhibit high-level gastric colonization in their respective host species albeit lacking *vacA* alleles [7,8]. 

The putative immunomodulatory and -suppressive properties of *H. pylori* VacA have been attributed to its profound effects on various types of immune cells. VacA is known to interact with myeloid cells as well as lymphocytes. Three types of interactions have been identified and studied in some detail. On the one hand, VacA has been reported to target professional phagocytes and to affect phagocytic killing of *H. pylori* by interfering with endocytic pathways. On the other hand, VacA is known to exploit the β2 integrin receptor to promote its uptake into human T-cells, where it interferes with T-cell proliferation, clonal expansion, and cytokine production. Finally, a new VacA-dependent mechanism of interference with normal functions of dendritic cells has recently been identified, which promotes the priming and differentiation of regulatory T-cells at the expense of effector T-cell differentiation. The evidence for all three immunomodulatory mechanisms is presented below in three dedicated sections. The implications of the findings for vaccine development, as well as for *H. pylori* eradication strategies and treatment decisions are discussed where appropriate, along with the known involvement/role of VacA in the (prevention of) extragastric diseases. Overall, this review is meant to give an up-to-date summary of the many facets of this important *H. pylori* colonization and persistence determinant that pertain to immunomodulation, and its molecular and cellular targets at the interface of the pathogen with the host immune system.

## 2. VacA Targets Phagocytes to Prevent Proper Phagosome Maturation, Antigen Processing and Presentation, Intracellular Killing, and Cytokine Production

**Figure 1:**
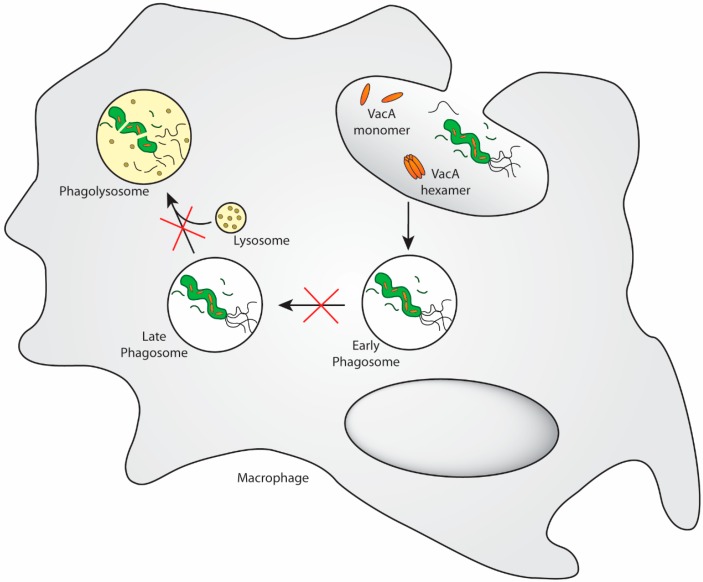
VacA prevents phagosome maturation in professional antigen-presenting cells. Most strains of *H. pylori* express the secreted virulence factors VacA to affect phagosome maturation and lysosome fusion. The receptor for VacA on myeloid cells is not known. It has been proposed that *H. pylori*-containing phagosomes fail to mature and instead resemble early endosomes that have resisted lysosome fusion; this process appears to be specific to type I strains and is dependent on VacA expression [12]. Treatment with recombinant IFN-γ allows phagocytes to overcome their block in phagosome maturation and to kill intracellular type I strains of *H. pylori.*

Multiple distinct mechanisms have been proposed to prevent or delay internalization of *H. pylori* by phagocytes, particularly macrophages, and to interfere with proper phagosome maturation and intracellular trafficking; several of these processes are believed to be dependent on VacA. *H. pylori* actively prevents or at least delays its uptake by macrophages [9,10]. Once it is inside the macrophages, *H. pylori* initially resides in single bacteria-containing phagosomes, which quickly fuse homotypically into so-called megasomes containing multiple viable bacteria and resembling giant multivesicular bodies [10]. This process of phagosome fusion appears to be specific for type I strains, *i.e.,* VacA- and Cag-PAI-positive strains [10], but could not be linked directly to either the Cag-PAI nor VacA using mutant strains in a follow-up study [11]. Whether *H. pylori* remains alive for extended periods of time in either murine and human monocytes or macrophages that have undergone phagosome fusion is not entirely clear [10,11]. Another study has shown that *H. pylori*-containing phagosomes fail to mature and instead resemble early endosomes that have resisted lysosome fusion; again, this process was specific to type I strains and, in this case, are also dependent on VacA expression (Figure 1) [12]. Treatment with recombinant IFN-γ enabled phagocytes to overcome their block in phagosome maturation and to kill intracellular type I strains of *H. pylori* [12]. A more recent study has followed up on the earlier observations using a co-infection model of murine bone-marrow-derived macrophages with *H. pylori* as well as *Lactobacillus acidophilus*, a commensal organism found throughout the intestinal tract that is known to promote Th1 polarization of T-cells. Whereas *L. acidophilus* exposure alone induced a Th1-polarizing and-promoting response of BM-derived macrophages characterized by high expression of IL-12 and IFN-β (such macrophages are also referred to as M1 macrophages), co-culture with wild-type *H. pylori* strongly reduced the expression of these cytokines [13]. A VacA-deficient mutant failed to interfere with *L. acidophilus*-induced cytokine expression in this manner. In a somewhat similar experimental setup using bone-marrow-derived dendritic cells (BM-DCs), VacA-proficient *H. pylori* effectively prevented the expression of DC maturation markers such as CD80 and CD86, as well as the expression and production of IL-12 upon LPS stimulation [2]. Whereas uninfected DCs readily upregulated CD80 and CD86 when treated with *E. coli* LPS, and produced large amounts of IL-12, TNF-α and IL-6, this TLR4-driven activation was strongly suppressed by wild-type *H. pylori* [2,14]. In contrast, VacA-deficient isogenic mutants in various *H. pylori* backgrounds failed to exhibit this suppressive phenotype [2], strongly implying VacA as an immunomodulatory factor targeting DCs, macrophages, or other professional phagocytes (Figure 1). The phenotype of macrophages or DCs that have been exposed to VacA-proficient, but not -deficient, *H. pylori* is reminiscent of semi-mature cells, characterized by high expression of MHC class II, but low expression of co-stimulatory molecules and a low or absent production of inflammatory and Th1-polarizing cytokines. The recently published observations on DCs [2] support and extend early work on VacA’s effects on antigen processing in B-cells [15]. To test the possibility that VacA alters antigen processing taking place in pre-lysosomal compartments, Molinari and colleagues used a model of antigen processing and presentation consisting of tetanus toxoid-specific human CD4^+^ T-cells stimulated by autologous antigen-pulsed Epstein–Barr virus-transformed B-cells. VacA was found to interfere with the proteolytic processing of the tetanus toxin and toxoid by B-cells and to specifically inhibit the antigen presentation by newly synthesized MHC class II, while leaving the presentation pathway dependent on recycling MHC class II unaffected [15]. In conclusion, although professional phagocytes and antigen-presenting cells, including B-cells, have been somewhat overlooked as potential VacA target cells, there is a considerable amount of definitive evidence in support of strong immunosuppressive effects—preventing Th cell-mediated immunity and persistent infection—on these cell types. 

## 3. VacA Blocks T-Cell Activation and Effector Functions

Experimental studies conducted in the course of almost two decades using various rodent models of *H. pylori* infection have shed light on the elements of the innate and adaptive immune system required for immune-mediated control of *H. pylori* infections; many such studies were designed to dissect the parameters and correlates of vaccine-induced protective immunity [16,17,18,19,20]. Whereas B-cells and antibodies are clearly dispensable for *H. pylori* control [18], it is now clear that CD4^+^ effector T-cells, and in particular Th1 and Th17-polarized T-effector cell subsets along with their signature cytokines, are critical for proper control of this infection [16,17,18,19,20]. Interestingly, the same T-cell subtypes have been implicated in initiating and promoting the immunopathological changes of the chronically infected gastric mucosa that manifest histologically as atrophic gastritis, compensatory epithelial hyperplasia, and intestinal metaplasia in experimentally infected animals and symptomatic human carriers [21,22]. VacA, as well as another persistence factor of *H. pylori*, the γ-glutamyl-transpeptidase, GGT, has been specifically implicated in the manipulation and inhibition of human T-cells. VacA inhibits T-cell proliferation by interfering with the T-cell receptor/interleukin-2 signaling pathway at the level of the Ca^2+^/calmodulin-dependent phosphatase calcineurin [23,24]. By preventing the nuclear translocation of the T-cell transcription factor NF-AT, VacA inhibits the subsequent transactivation of T-cell-specific immune response genes [23,24]. Follow-up studies have since identified β2 integrin (CD18) as the receptor for VacA on human T-cells [25]; β2 integrin heterodimerizes with CD11a on T-cells to form the transmembrane receptor LFA-1 (lymphocyte function-associated antigen-1). *H. pylori* exploits the recycling of LFA-1 for VacA uptake [25] in a manner that depends on protein kinase C-mediated serine/threonine phosphorylation of the β2 integrin cytoplasmic tail [26]. The second *H. pylori* virulence determinant implicated in T-cell inhibition, the GGT enzyme, appears to block T-cell function in a similar manner [27,28]. Like VacA, GGT is a secreted factor that most if not all isolates of *H. pylori* encode and express. GGT blocks proliferation of T-cells through a mechanism that involves the inhibition of cyclin-dependent kinase activity in the G1 phase of the cell cycle by the disruption of the Ras signaling pathway [27,28]. 

## 4. VacA Promotes the Differentiation of Regulatory T-Cells over Effector T-Cells and Thereby Ensures Persistent *H. pylori* Infection

In line with the ability of VacA-proficient strains of* H. pylori* to retain professional antigen-presenting cells in a semi-mature state (*i.e.*, high MHC class II, low CD80/CD86, low pro-inflammatory cytokine production), several reports have focused on the downstream consequences for T-cell priming. VacA-deficient mutants and purified VacA were both used to address the type of T-cell responses, the predominant cytokines produced, and the consequences of these immunological parameters for persistent *H. pylori* colonization and the T-cell-mediated responses to other (non-*Helicobacter*-derived) antigens. The lower levels of colonization of VacA mutants relative to their parental wild type was found to correlate with higher numbers of Th1 and Th17 cells, higher expression of IFN-γ and IL-17 by restimulated mesenteric lymph node (MLN) single cell preparations, and lower numbers of FoxP3^+^ CD25^+^ regulatory T-cells in MLNs [2]. The difference in T helper cell responses, which are biased towards regulatory T-cell responses in the presence of VacA, was attributed to VacA’s effects on DCs. DCs that were immunomagnetically purified based on their CD11c expression from the MLNs of wild-type-infected mice induced FoxP3 and CD25 expression in co-cultured naive CD4^+ ^T-cells *ex vivo* [2]. This was not observed with DCs from uninfected animals or from mice infected with a VacA mutant [2]. The defect of the VacA mutant in tolerizing dendritic cells *in vivo* was also confirmed in *in vitro* settings, where BM-DCs co-cultured with the wild-type bacteria, but not with various VacA mutants in several strain backgrounds, induced FoxP3 and CD25 expression in co-cultured T-cells [2]. The semi-mature phenotype of DCs thus correlates with their ability to induce Treg differentiation, as has been described in many other experimental settings [29]. The long-term consequences of VacA deficiency are all related to the overshooting effector T-cell responses seen with these mutants, as they cause more severe inflammation, atrophic gastritis, intestinal metaplasia, and epithelial hyperplasia than their corresponding wild-type strain [2]. This phenotype in the mouse model is striking given the well known toxigenic activity of VacA on human mucosal epithelial cells, but is well in line with its immunosuppressive and immunomodulatory properties.

**Figure 2:**
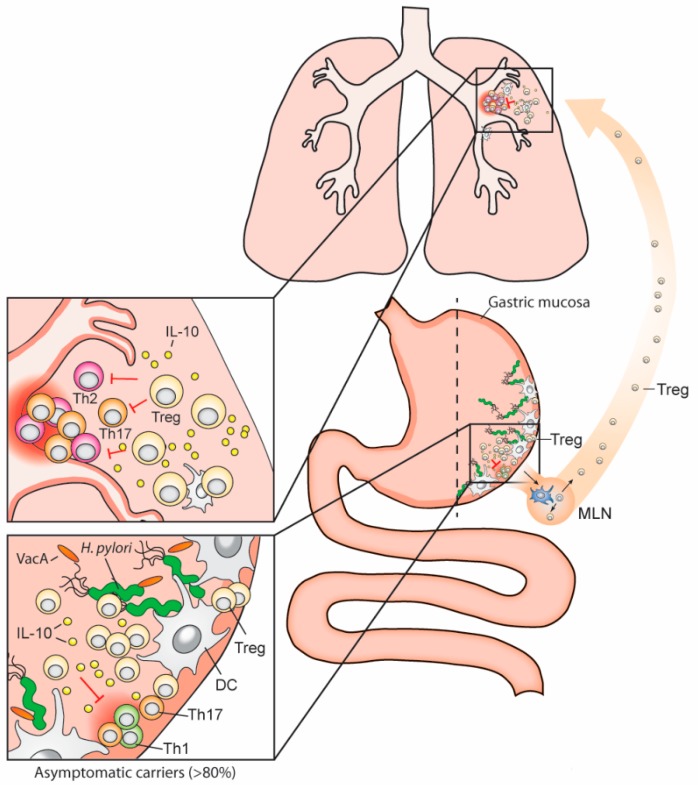
*H. pylori* exerts immunomodulatory effects at extragastric sites. *H. pylori* exclusively inhabits the gastric mucosa but has systemic immunomodulatory effects that manifest in the airways, lower gastrointestinal tract, and possibly the skin. Gastric tissue-resident DCs presumably sample *H. pylori* antigens locally at the site of infection and subsequently migrate to the stomach-draining and mesenteric lymph nodes (MLNs), where they prime T-effector and regulatory T-cell responses. VacA is believed to skew this process towards Treg differentiation [2]. MLN-derived, *H. pylori*-induced Tregs enter the circulation and, due to cross-talk between the GI tract and lung immune systems, may accumulate in both the gastric mucosa and the lung. According to current models, allergen-specific Th2 cells in the lung are suppressed by *H. pylori*-induced Tregs via soluble mediators (such as IL-10) and contact-dependent mechanisms (reviewed in [43]). Tregs further suppress Th1 and Th17 responses directed against *H. pylori* in the gastric mucosa [30,44]. DC: dendritic cell; Th1/2/17: T-helper cell subsets; Treg: regulatory T-cell. Please note that, due to space restrictions, the MLN shown here is not in its correct anatomical location, which would be in the mesentery, linking the intestines to the abdominal wall.

*H. pylori*-VacA-induced Tregs not only ensure persistent *H. pylori* infection in the mouse model [30], but also drive immune tolerance to non-related T-cell antigens. This has been shown in models of allergen-induced asthma using ovalbumin or house dust mite allergens [2]. The induction of Tregs with suppressive activity against allergen-specific T-cell responses depends on VacA, as VacA mutants fail to suppress allergen-induced asthma (Figure 2) [2]. Furthermore, the adoptive transfer of MLN-derived Tregs from wild-type-infected donors to naive recipients protects the recipients from allergic asthma; this Treg-dependent protection is not seen with cells from VacA mutant-infected donors [2]. The immunomodulatory activity of VacA was further shown to be retained in purified protein from culture supernatants of *H. pylori* [31]. The s1m1 variant of VacA is readily secreted by bacteria growing in liquid culture media, from which it can be purified in its oligomeric form [32]. Administration of several doses of VacA protects efficiently against allergen-induced asthma, especially if the protein is provided in the neonatal tolerance window [31]. This time frame constitutes a period in both mice and humans in which immune tolerance to antigens is readily established [33]; therefore, it is perhaps not surprising that VacA acts most potently during this time [31]. Active tolerization against allergens using VacA requires its interaction with DCs, as mouse strains lacking IL-10 expression in the DC compartment cannot be tolerized with VacA [31]. These results again speak in favor of selective targeting of VacA to DCs (or at least CD11c-positive myeloid cells), where it exerts tolerance-promoting activity. Further work is required to definitively show that VacA interacts with DCs *in vivo*, either in the gastric mucosa or the draining lymph nodes, and that VacA-tolerized DCs are functionally different from naive DCs. 

Several studies have emphasized the role of (VacA-induced) Tregs in the *H. pylori*/host interaction in both humans and experimentally infected mice. The experimental depletion of Tregs facilitates clearance of *H. pylori* in infected animals [30,34,35] and enhances vaccine-induced protective immunity in vaccinated mice [36]. The Treg-driven persistence of *H. pylori* requires T-cell-specific expression of IL-10; in fact, IL-10^−/−^ mice and a strain lacking IL-10 expression exclusively in the CD4^+^ T-cell compartment are capable of spontaneously controlling experimental infections [30,37,38,39]. The efficient control or even clearance of *H. pylori* in Treg-depleted animals invariably comes at the price of enhanced gastric immunopathology (gastritis and epithelial preneoplastic changes such as atrophy and intestinal metaplasia). Interestingly, an analogous observation has been reported for human carriers, which either accumulate large numbers of IL-10-producing, *H. pylori*-specific Tregs and are colonized heavily (asymptomatic carriers), or develop gastric ulcers because their Treg response is inadequate [40]. In summary, experimental studies in infected animals and observational studies in humans support a critical role of Tregs in maintaining a benign interaction between *H. pylori* and its host, preventing bacterial clearance on the one hand, and limiting immunopathology on the other.

Given the well-documented immunomodulatory activity of VacA on T-cells, myeloid cells, and potentially other cells of the mammalian immune system, it comes as no surprise that vaccine development efforts have focused on this molecule as a promising target of vaccination. In fact, recombinantly produced VacA, along with two other highly immunogenic *H. pylori* proteins, CagA and NAP, have not only been extensively tested in murine vaccine models [41] but also in humans. A clinical phase I trial assessing the safety and immunogenicity of a vaccine consisting of the three recombinant proteins, given intramuscularly with aluminium hydroxide as an adjuvant to noninfected healthy subjects, concluded that this experimental vaccine demonstrated satisfactory safety and immunogenicity profiles and produced both humoral immunity and antigen-specific T-cell memory [42]. However, efficacy has not been documented in humans for a VacA-containing vaccine. The potential of VacA as a vaccine antigen and its documented activity against allergic asthma in experimental models of disease prevention clearly warrant further research into the immunomodulatory properties of this multifunctional persistence determinant of *H. pylori*.

## 5. Conclusions

Despite being better known for its vacuolating and cytotoxic activity, VacA has also recently received considerable attention for its immunomodulatory properties. A range of effects on several types of immune cells has been attributed to VacA *in vitro* and *in vivo*; the protein’s ability to insert into lipid bilayers to form anion-selective channels appears to be a prerequisite of both its cytotoxic and immunomodulatory activity. Whether and how VacA targets immune cells in the gastric mucosa remains to be determined; an *in vivo* tracking system using GFP- or otherwise tagged VacA would be a highly useful tool in this respect. It is conceivable that VacA secreted from bacteria residing in the gastric mucus, or in close proximity to gastric epithelial cells, is readily sampled by intraepithelial DCs and macrophages that extend protrusions into the gastric lumen. It is less straightforward to envision VacA targeting T-cells, which reside exclusively in the lamina propria. A direct interaction of VacA with T-cells would require disruption of epithelial tight junctions and the subsequent loss of epithelial barrier function, processes that are known to occur during *H. pylori* infection of polarized monolayers [45,46]. More evidence is needed from both naturally infected humans hosts as well as experimental animals to better understand the immunological consequences of VacA-positive *H. pylori* infection. In particular, more work is needed to clarify the differential cytotoxic and immunomodulatory activity of s1i1m1 VacA relative to its less toxigenic counterparts encoded by other alleles. Recent work on *H. pylori* strains in which the endogenous allele was exchanged for one of two alternative, naturally occuring alleles [3] to generate isogenic strains, differing only in the type of VacA they produce, represents a step in the right direction and model for future work. The identification of a (putative) VacA receptor on immune cells other than T-cells is also of great importance, as dendritic cells and macrophages likely represent the first line of defence (and first target cell of VacA) encountered by *H. pylori*. A better understanding of VacA’s target cells and mechanism of action will facilitate its targeting and exploitation for new treatment and prevention strategies.
